# Development and validation of prediction model for older adults with cognitive frailty

**DOI:** 10.1007/s40520-023-02647-w

**Published:** 2024-01-28

**Authors:** Jundan Huang, Xianmei Zeng, Hongting Ning, Ruotong Peng, Yongzhen Guo, Mingyue Hu, Hui Feng

**Affiliations:** 1 Xiangya School of Nursing, Central South University, Changsha, 410013 Hunan China; 2 Oceanwide Health Management Institute, Central South University, Changsha, China; 3 National Clinical Research Center for Geriatric Disorders, Xiangya Hospital, Central South University, Changsha, China

**Keywords:** Cognitive frailty, Cognitive impairment, Prediction model, Older adults

## Abstract

**Objective:**

This study sought to develop and validate a 6-year risk prediction model in older adults with cognitive frailty (CF).

**Methods:**

In the secondary analysis of Chinese Longitudinal Healthy Longevity Survey (CLHLS), participants from the 2011–2018 cohort were included to develop the prediction model. The CF was assessed by the Chinese version of Mini-Mental State Exam (CMMSE) and the modified Fried criteria. The stepwise regression was used to select predictors, and the logistic regression analysis was conducted to construct the model. The model was externally validated using the temporal validation method via the 2005–2011 cohort. The discrimination was measured by the area under the curve (AUC), and the calibration was measured by the calibration plot. A nomogram was conducted to vividly present the prediction model.

**Results:**

The development dataset included 2420 participants aged 60 years or above, and 243 participants suffered from CF during a median follow-up period of 6.91 years (interquartile range 5.47–7.10 years). Six predictors, namely, age, sex, residence, body mass index (BMI), exercise, and physical disability, were finally used to develop the model. The model performed well with the AUC of 0.830 and 0.840 in the development and external validation datasets, respectively.

**Conclusion:**

The study could provide a practical tool to identify older adults with a high risk of CF early. Furthermore, targeting modifiable factors could prevent about half of the new-onset CF during a 6-year follow-up.

**Supplementary Information:**

The online version contains supplementary material available at 10.1007/s40520-023-02647-w.

## Background

The global population of older adults was approximately 1 billion in 2019 and will increase to 1.4 billion by 2030 [1]. Ageing contributes to many chronic conditions, such as cognitive impairment and frailty, which have become increasingly significant public health problems [2, 3]. Frailty is influenced by multidomain factors, including age, sex, risk of malnutrition, and chronic diseases, as well as disability [4, 5]. Frailty and cognitive impairment interact in the ageing process, increasing the risk of adverse outcomes [6] such as dementia, disability, and mortality, but they have historically been studied separately. Consequently, the International Consensus Group from the International Academy of Nutrition and Aging (IANA) and the International Association of Gerontology and Geriatrics (IAGG) have proposed that cognitive frailty (CF) is a clinical condition characterized by the occurrence of both physical frailty and cognitive impairment and in the absence of dementia diagnosis [7]. CF may contribute to a higher risk of adverse outcomes than healthy older adults or those with physical frailty or cognitive impairment alone [8–10]. A meta-analysis indicated that the pooled prevalence of CF among community-dwelling older adults was 9% [11].

In order to identify individuals at high risk of CF and to facilitate the implementation of appropriate preventive measures and interventions [12–14], some prediction models have been developed [15–17]. There were some limitations in existed CF prediction models, such as selecting predictors based on univariable analysis and lacking calibration and external validation [15]. For instance, Peng et al. [16] developed and internally validated a prediction model for diagnosing CF in elderly Chinese patients with multimorbidity, incorporating non-traditional factors. The final model included nine predictors, yielding an area under the curve (AUC) of 0.9908. However, the predictors of this model were chosen through univariable analysis, and no external validation was performed, potentially compromising the prediction performance of the final model. The accessibility of predictors was another question. Sargent et al. [17] constructed a prediction model for CF using 32 single nucleotide polymorphisms (SNPs) and 155 protein biomarkers, achieving a model AUC of 0.88. However, the high cost and limited accessibility of predictors restricted the generalizability and applicability of models.

Therefore, we aimed to develop and validate a CF prediction model among older adults using easily obtainable predictors and adhering strictly to the Transparent Reporting of a Multivariable Prediction Model for Individual Prognosis or Diagnosis (TRIPOD) statement [18]. The study’s findings might be more helpful for primary medical staffs in the early detection of CF.

## Methods

This study was a secondary analysis of the Chinese Longitudinal Healthy Longevity Survey (CLHLS) and strictly followed the TRIPOD statement [18]. The detailed information is given in Supplementary Table 1.

### Source of data and participants

Participants were chosen from CLHLS, a prospective multicenter cohort study covering about 113,000 older adults in China. Surveys of the CLHLS began in 1998 and were conducted every three years, with eight surveys conducted from 1998 to 2018. We selected the 2011–2018 cohort for model development in this study. The included criteria were the followings: (1) older adults aged 60 years and over; (2) absence of CF at baseline; and (3) at least one follow-up.

Exclusion criteria encompassed individuals with dementia and cancer at baseline. The study excluded individuals with dementia to emphasize the focus on CF, involving reversible cognitive impairment, particularly mild cognitive impairment, as opposed to irreversible dementia [19]. Additionally, the exclusion of individuals diagnosed with cancer at the baseline was aimed at minimizing potential attrition rates over the extended 6-year follow-up duration due to their shorter life expectancy.

Additionally, we used the 2005–2011 cohort of CLHLS for external validation, and the inclusion and exclusion criteria were consistent with the development cohort.

### Assessment of cognitive frailty

In this study, CF was defined as the coexistence of cognitive impairment and physical frailty. Cognitive impairment was measured by the Chinese version of Mini-Mental State Exam (CMMSE) [20]. The CMMSE consists of 24 items, scored from 0 to 30, with higher scores indicating better cognitive function. The definition of cognitive impairment was corrected by the educational levels [21, 22]. Participants had been diagnosed cognitive impairment with CMMSE score between 16 to 19 for those illiterate people, 20 to 22 for those with elementary school education, and 23 to 26 for those with a middle school education or higher score.

Physical frailty was evaluated by the modified Fried criteria which consisted of five domains: exhaustion, shrink, weakness, low mobility, and inactivity [23]. Each domain was assessed with a binary response (yes or no). Individuals were categorized as frailty if they reported three or more domains as "yes". Exhaustion was defined if the participants answered “always”, “often” or “sometimes” to the question “I felt old and useless”. Shrink was defined as body mass index (BMI) < 18.5 kg/m^2^, calculated by dividing the weight by the square of the height. Weakness was defined as the participants’ self-reported inability to lift a bag weighing 5 kg. Low mobility was defined as the participants’ self-reported inability to walk one km. Inactivity was defined if participants reported that they engaged in activities once a week or less. Activities included housework, outside activity, gardening, keeping a pet, livestock breeding, playing cards or mah-jong, and social activity.

### Candidate predictors

Candidate predictors were selected based on previous studies [24–28], medical knowledge and data available in the database. A total of 12 candidate predictors were chosen, and the detailed information is shown in Table 1. The physical disability was measured using the instrumental activity of daily living (IADL) and the basic activity of daily living (BADL), and it was defined if any item of IADL or BADL was judged as dependence [29]. Most variables were self-reported by the older adults or their families except BMI.

**Table 1:** Details of candidate predictors at baseline in the development and validation datasets

Candidate predictors	Development dataset (n = 2420)			Validation dataset (n = 3512)		
	Non-CF (n = 2177)	CF (n = 243)	*P *value	Non-CF (n = 3108)	CF (n = 404)	*P* value
Age (year), median (IQR)	75.00 (71.00, 82.00)	86.00 (79.50, 93.00)	** < 0.001**	74.00 (69.00, 82.00)	85.00 (77.50, 90.00)	** < 0.001**
Sex, n (%)			** < 0.001**			** < 0.001**
Male	1138 (52.3)	77 (31.7)		1535 (49.4)	130 (32.2)	
Female	1039 (47.7)	166 (68.3)		1574 (50.6)	274 (67.8)	
Residence, n (%)			0.317			0.117
City	1248 (57.3)	132 (54.3)		573 (18.4)	82 (20.3)	
Town	294 (13.5)	29 (11.9)		596 (19.2)	91 (22.5)	
Rural	635 (29.2)	82 (33.7)		1939 (62.4)	231 (57.2)	
Education, n (%)			** < 0.001**			** < 0.001**
Illiteracy	961 (44.1)	168 (69.1)		1529 (49.2)	311 (77.0)	
Primary school	873 (40.1)	60 (24.7)		1171 (37.7)	66 (16.3)	
Secondary school or above	343 (15.8)	15 (6.2)		408 (13.1)	27 (6.7)	
QOL, n (%)			0.141			**0.025**
Very good	388 (17.8)	45 (18.5)		450 (14.5)	57 (14.1)	
Good	947 (43.5)	92 (37.9)		1407 (45.3)	194 (48.0)	
General	755 (34.7)	90 (37.0)		1080 (34.7)	122 (30.2)	
Bad	87 (4.0)	16 (6.6)		171 (5.5)	31(7.7)	
Exercise, n (%)	2067 (94.9)	215 (88.5)	** < 0.001**	2924 (94.1)	362 (89.6)	**0.001**
Physical disability, n (%)	296 (13.6)	127 (52.3)	** < 0.001**	458 (14.7)	193 (47.8)	** < 0.001**
Marriage, n (%)			** < 0.001**			** < 0.001**
Married	1322 (60.7)	75 (30.9)		1774 (57.1)	106 (26.3)	1880 (53.5)
Widowed	827 (38.0)	167 (68.7)		1308 (42.1)	298 (73.8)	1606 (45.7)
Unmarried	28 (1.3)	1 (0.4)		26 (0.8)	0 (0.0)	26 (0.7)
BMI, n (%)			** < 0.001**			** < 0.001**
Underweight	283 (13.0)	76 (31.3)		1654 (53.2)	175 (43.3)	
Normal	1235 (56.7)	116 (47.7)		1043 (33.6)	189 (46.8)	
Overweight	487 (22.4)	37 (15.2)		317 (10.2)	28 (6.9)	
Obesity	172 (7.9)	14 (5.8)		94 (3.0)	12 (3.0)	
Sleep, n (%)			**0.018**			**0.021**
Good	1397 (64.2)	138 (56.8)		2099 (67.6)	258 (63.9)	
General	535 (24.6)	64 (26.3)		691 (22.2)	114 (28.2)	
Poor	245 (11.3)	41 (16.9)		318 (10.2)	32 (7.9)	
Smoke, n (%)	491 (22.6)	39 (16.0)	**0.025**	2263 (72.8)	349 (86.4)	** < 0.001**
Drink, n (%)	483 (22.2)	33 (13.6)	**0.002**	2326 (74.8)	337 (83.4)	** < 0.001**

Bold values represent statistically significant results

*CF* cognitive frailty, *IQR* interquartile range, *n* number, *QOL* quality of life, *BMI* body mass index

### Sample size

According to the Tool to Assess Risk of Bias and Application of Prediction Model Studies (PROBAST) [30], a minimum of 20 events per variable (EPVs) was suggested for model development and at least 100 participants with the outcome for model validation to minimize overfitting. More than 240 older adults with CF must be in the development cohort, and there should be at least 100 participants with CF in the validation cohort.

### Missing data

The missing data were dealt with multivariate imputation by chained equation (MICE) if the missing data in each variable were random and accounted for less than 50% [31]. Five imputations were generated using multiple chains. The dataset with the lowest Akaike Information Criterion (AIC) value was chosen.

### Statistical analysis

The continuous data were presented as mean with standard deviation (SD) if the data were normally distributed, verified using histogram and Kolmogorov–Smirnov test. Non-normally distributed data were presented as median with interquartile range (IQR). The categorical data were presented as the number with proportions. BMI was classified into four categories [32]. More details can be found in Supplementary Table 2. The categarical data were analyzed using the chi-square test (for theoretical frequencies T ≥ 5) or continuity correction for theoretical frequencies (for theoretical frequencies T < 5). For continuous data, an independent samples t-test was utilized for normally distributed data; otherwise, the non-parametric Wilcoxon rank-sum test was applied.

For model development, we used stepwise binary logistic regression analysis to select predictors and develop a model with the lowest AIC value. For internal validation, we evaluated the model’s performances by discrimination and calibration. The discrimination, often measured by AUC, was the extent to distinguish those at higher or lower risk of having an event [33]. The discrimination was evaluated as acceptable with AUC of 0.7–0.8, excellent with AUC of 0.8–0.9, and outstanding with AUC of 0.9 [34]. The calibration evaluated the conformity of predicted and actual risks, measured by the calibration plot. The calibration plot represented the predictive probability on the x-axis and the observed probability on the y-axis. A 45-degree line in the calibration plot suggested perfect prediction [35].

To test the robustness of the model, we conducted several sensitivity analysis. First, we excluded the BMI from the model to investigate the effect of incorporation bias. Then, we excluded participants with missing data to explore selection or attrition bias.

Additionally, we calculated the population attributable fraction (PAF) for the proportion of people exposed to risk factors. The formula is as follows:

$$\begin{array}{c}PAF=\frac{\sum_{i=1}^{n}{P}_{i}R{R}_{i}-\sum_{i=1}^{n}{P}_{i}{\prime}R{R}_{i}}{\sum_{i=1}^{n}{P}_{i}R{R}_{i}}\end{array}$$

where the P_i_ is the population proportion at exposure level i; P_i_^’^ is the counterfactual or ideal level of exposure; RR_i_ is the risk ratio at exposure level i, and n is the number of exposure levels.

All statistical analyses were performed using the R version 4.1.3 software with major packages of mice, MASS, regplot, rms, and pROC.

### Internal and external validation

The model was internally validated using the bootstrap resampling for 1000 times method. To check the external validation, we performed the temporal validation using data from the CLHLS 2005–2011 cohort. There was no difference between the development and validation datasets in setting, CF assessment, and predictors.

## Results

### Participants

**Fig. 1:**
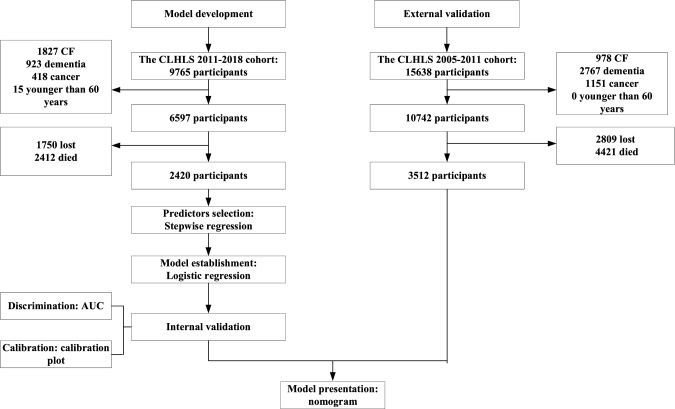
Flow diagram of study design

The development dataset consisted of 2420 participants, among whom 243 older adults (10.04%) were observed to have CF during a median follow-up period of 6.91 years (IQR: 5.47–7.10 years). In the external validation dataset, a final analysis of 3512 participants was conducted, revealing 404 older adults with CF. The detailed flowchart of the study population is shown in Fig. 1. The characteristics of the participants are shown in Table 1. Compared with those having complete data, individuals with missing data showed no statistical difference in all variables except physical disability (*p* < 0.001). The characteristics of the participants between those with missing data and complete data are shown in Supplementary Table 3.

### Predictors selection and model development

According to the lowest AIC value, the stepwise binary logistic regression analysis was used for predictors selection and model development. Finally, six predictors were included to develop the model (i.e., age, sex, residence, BMI, exercise, and physical disability). The importance of the six predictors was ranked as follows: age, physical disability, BMI, sex, residence, and exercise, according to difference value between full model pseudo-R^2^ and models with any excluded variable. And the details are shown in the Supplementary Table 4. Table 2 displays the model’s coefficient and odds ratio (OR).

**Table 2:** The coefficient and odds ratio of the CF prediction model

Predictors	Coefficient (95% CI)	P-value	OR (95%CI)
Age	**0.09 (0.08, 0.11)**	** < 0.0001**	**1.09 (1.08, 1.12)**
Sex			
Male	Ref	NA	Ref
Female	**0.33 (0.02, 0.64)**	**0.0388**	**1.09 (1.08, 1.11)**
BMI			
Underweight	**0.85 (0.49, 1.21)**	** < 0.0001**	**2.34 (1.63, 3.37)**
Normal	Ref	NA	Ref
Overweight	– 0.08 (– 0.52, 0.32)	0.6810	0.91 (0.59, 1.39)
Obesity	0.13 (– 0.53 0.72)	0.6897	1.14 (0.59, 2.06)
Residence			
City	Ref	NA	Ref
Town	0.18 (– 0.30, 0.64)	0.4461	1.20 (0.74, 1.90)
Rural	**0.34 (0.01, 0.67)**	**0.0438**	**1.41 (1.01, 1.93)**
Exercise	– 0.49 (– 0.99, 0.03)	0.0568	0.61 (0.37, 1.03)
Physical disability	**1.41 (1.10, 1.72)**	** < 0.0001**	**4.11 (3.01, 5.60)**

Bold values represent statistically significant results

*OR* odds ratio, *CI* confidence interval, *Ref* reference, *NA* not applicable, *BMI* body mass index.

### Model performance

The discrimination of CF prediction model was excellent [34], indicated by an AUC of 0.830 (95% CI 0.802–0.858). The model's cut-off point was – 2.271, with a specificity of 0.844 and a sensitivity of 0.907. The calibration plot revealed a high level of consistency between the predicted risk of CF and the observed risk.

**Fig. 2:**
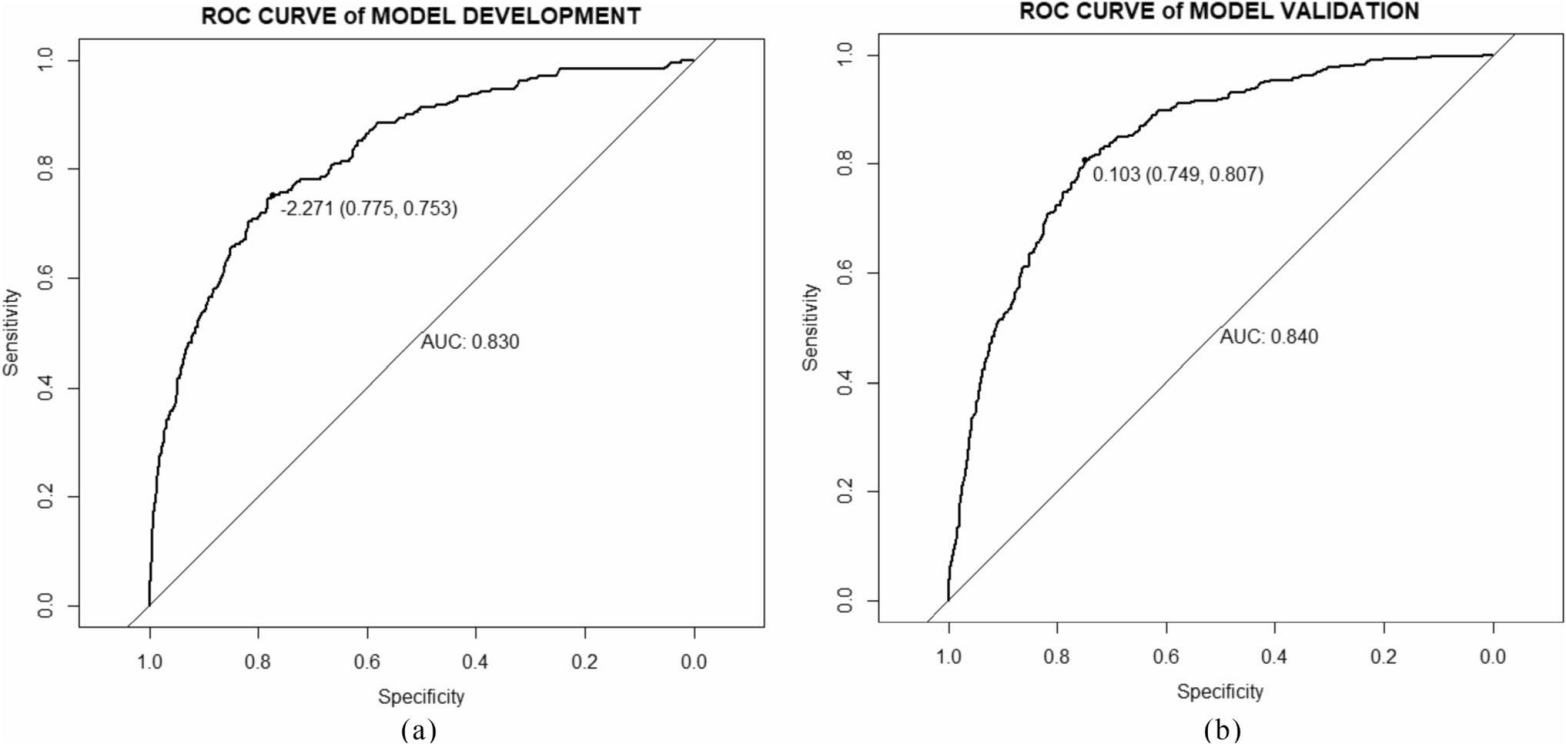
AUC of the prediction model in the development (**a**) and validation cohort (**b**)

**Fig. 3:**
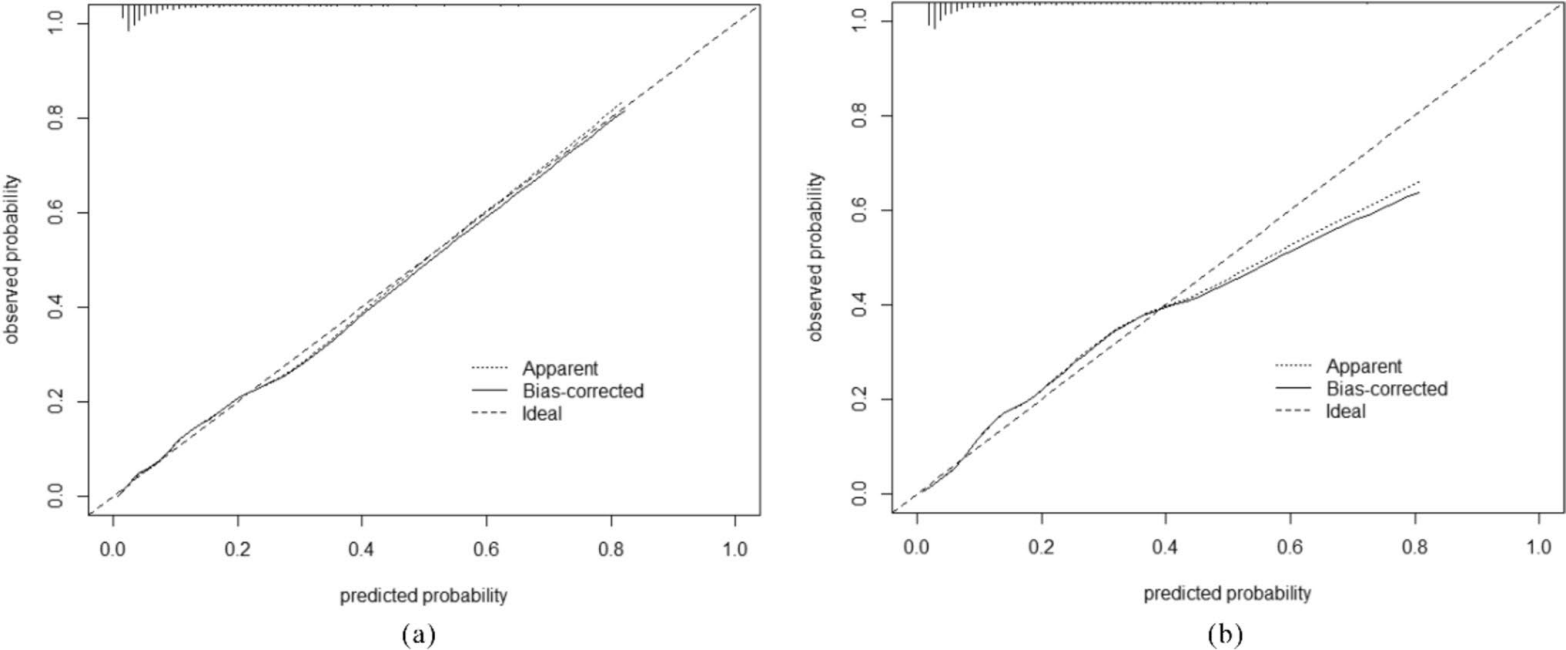
The calibration plot of the prediction model in internal (**a**) and external validation (**b**)

In the external validation, the model performed better, with a higher discrimination AUC of 0.840 (95% CI 0.820–0.860) and a well-fitted calibration curve. Figures 2 and 3 display the prediction performance.

### Model presentation

The nomogram was employed to illustrate the model, allowing the calculation and summation of points based on participants' characteristics. The total score was calculated by summing the values of each predictor for an older adult. By drawing a line down corresponding to this total score, one could obtain the predicted probability of CF (Supplementary Fig. 1).

### Sensitivity analysis

After excluding BMI from the model, the AUC slightly attenuated, with AUC of 0.824 (95% CI 0.795–0.852) (Supplementary Fig. 2). After excluding participants with missing data, the AUC of the model was 0.836 (95% CI 0.786–0.888) (Supplementary Fig. 3), which proved that the model was robust.

### Population attributable fraction

Among the six predictors, three were modifiable: BMI, exercise, and physical disability. The results of PAF suggested that targeting the three modifiable predictors would theoretically decrease 53.79% of the 6-year risk of CF.

## Discussion

We developed a CF prediction model using multivariable logistic regression with the CLHLS database. Predictors considered in this model encompassed age, sex, residence, exercise, BMI, and physical disability. The internal and external validation results exhibited that the model was with excellent discriminative ability and calibration.

Consistent with other studies [28, 36], aging and female were risk factors of CF, while living in rural areas posed a higher risk than living in the city settings. Aging has been identified as a catalyst for chronic inflammation, and was often correlated with compromised physical functionality and diminished muscle mass. This augmented inflammatory state could have significant ramifications on cognitive function, particularly memory and executive function, thereby elevating the risk of CF [37]. Regarding the gender, the increased risk of CF in women may be related to hormonal factors, compared with men. Studies suggested that the decrease in testosterone and other androgens might be linked to the progression of frailty and cognitive decline [38, 39]. Testosterone plays a protective role in cognition by promoting synaptic plasticity in the hippocampus and regulating the accumulation of amyloid-beta proteins [39]. Additionally, a reduction in testosterone levels can lead to the decline in muscle mass [38]. Rural older residents in mainland China had a higher risk of CF, possibly due to unequal urban–rural resources, lower income, low education levels among rural inhabitants, and limited access to healthcare services and insurance [40].

Our model showed that underweight, physical inactivity and physical disability increased the risk of CF. These findings were consistent with previous studies [41–43]. Low BMI and physical inactivity can increase the content of metabolites and inflammatory cells, affecting structure in the brain [44–46]. Additionally, previous studies showed that inflammation was significantly associated with poor physical function and muscle strength in older adults, thereby leading to an increased risk of CF [47, 48]. The nutritional and exercise intervention could effectively improve muscle strength and physical function among older adults, thereby reducing the risk of CF [49, 50]. One study showed that participants in the physical activity group had 21% lower odds of worsening CF over 24 months than those in the health education group [51]. Nevertheless, the best doses of type, intensity, and timing of exercise for preventing CF still need to be explored [52]. Despite the lack of significant impact on CF, exercise remained a constituent of the final model. The predictors were computed following the AIC methodology and were informed by scientific insights, underscoring the pivotal role of exercise in CF [12, 52].

### Strengths

Our study had some strengths. Firstly, the participants were representative because they were from a large population-based cohort. Secondly, the predictors included were non-invasive, low-cost, and easy to obtain, so the prediction model could be used in the primary care settings [53]. Thirdly, we performed external validation for portability and generalization, and the model displayed excellent discrimination and calibration in external validation. Lastly, we calculated the PAF to explore how modifiable risk factors contribute to CF.

### Limitations

Our study also had some limitations. Firstly, most predictors were self-reported by older adults, potentially introducing information bias. Nevertheless, self-reported predictors were more easy-to-obtain and practical [54]. Secondly, some critical predictors were not included due to data limitations, such as depression [28], likely impacting the prediction performance. Thirdly, BMI, a part of the CF assessment, was selected as a predictor, which might introduce incorporation bias and optimist estimates of model performance [55]. However, the sensitivity analysis indicated that after removing the BMI, the prediction model maintained excellent performance. Therefore, the incorporation bias may have a negligible effect on the model performance.

### Implications and clinical practice

This study presents some insights for future research. Firstly, the selection of predictive factors, encompassing easily accessible, non-invasive, and cost-effective variables, plays a pivotal role in prediction models applicable to clinical practice, especially within community healthcare and diverse clinical settings. Subsequent research endeavors should consider integrating addition predictive factors that share the accessibility, non-invasiveness, and cost-effectiveness criteria. Secondly, our study is grounded in the application of the logistic regression method for model development. Future research could explore alternative methodologies, such as machine learning techniques, to foster the evolution of predictive models. Lastly, the model's foundation is rooted in the Chinese population, prompting the necessity to examine its transferability to other demographics. This calls for comprehensive validation in diverse populations to establish its broader applicability in the future.

This study developed a prediction model for CF based on the characteristics of the Chinese population, utilizing practical, non-invasive, cost-effective, and easily obtainable variables. It can be applied in secondary prevention, enabling early identification, diagnosis, and treatment of CF. In tertiary disease prevention, the predictive model can be used to forecast recurrence, reduce mortality and disability [56]. Furthermore, it can provide community staffs with insights into the progression of CF in the older adults, allowing the identification of potential contributing factors for tailored preventive interventions [54].

## Conclusion

The CF prediction model, following the TRIPOD statement, has been established and validated for older adults. It integrates six easily obtainable predictors and demonstrates excellent prediction performance. This model helps healthcare practitioners and nurses to identify older adults at a heightened risk of CF development over a six-year period and intervene proactively.

### Supplementary Information

Below is the link to the electronic supplementary material.

Supplementary file1 (DOCX 215 KB)

## Data Availability

The CLHLS data can be obtained by applying on the website. https://opendata.pku.edu.cn/dataset.xhtml?persistentId=doi:10.18170/DVN/WBO7LK&version=2.0.
