# Supplementary material for: Development and validation of prediction model for older adults with cognitive frailty
Source: Aging Clin Exp Res. 2024 Jan 28;36(1):8. doi: 10.1007/s40520-023-02647-w (PMC10822804; doi:10.1007/s40520-023-02647-w)

**Supplementary table 1.** Reporting checklist for prediction model development/validation.

|  |  | Reporting Item | Page number |
| --- | --- | --- | --- |
| **Title** |  |  |  |
|  | [#1](https://www.goodreports.org/reporting-checklists/tripod/info/#1) | Identify the study as developing and / or validating a multivariable prediction model, the target population, and the outcome to be predicted. | 1 |
| **Abstract** |  |  |  |
|  | [#2](https://www.goodreports.org/reporting-checklists/tripod/info/#2) | Provide a summary of objectives, study design, setting, participants, sample size, predictors, outcome, statistical analysis, results, and conclusions. | 1 |
| **Introduction** |  |  |  |
|  | [#3a](https://www.goodreports.org/reporting-checklists/tripod/info/#3a) | Explain the medical context (including whether diagnostic or prognostic) and rationale for developing or validating the multivariable prediction model, including references to existing models. | 2-3 |
|  | [#3b](https://www.goodreports.org/reporting-checklists/tripod/info/#3b) | Specify the objectives, including whether the study describes the development or validation of the model or both. | 3 |
| **Methods** |  |  |  |
| Source of data | [#4a](https://www.goodreports.org/reporting-checklists/tripod/info/#4a) | Describe the study design or source of data (e.g., randomized trial, cohort, or registry data), separately for the development and validation data sets, if applicable. | 3-4 |
| Source of data | [#4b](https://www.goodreports.org/reporting-checklists/tripod/info/#4b) | Specify the key study dates, including start of accrual; end of accrual; and, if applicable, end of follow-up. | 4 |
| Participants | [#5a](https://www.goodreports.org/reporting-checklists/tripod/info/#5a) | Specify key elements of the study setting (e.g., primary care, secondary care, general population) including number and location of centres. | 4 |
| Participants | [#5b](https://www.goodreports.org/reporting-checklists/tripod/info/#5b) | Describe eligibility criteria for participants. | 4 |
| Participants | [#5c](https://www.goodreports.org/reporting-checklists/tripod/info/#5c) | Give details of treatments received, if relevant | NA |
| Outcome | [#6a](https://www.goodreports.org/reporting-checklists/tripod/info/#6a) | Clearly define the outcome that is predicted by the prediction model, including how and when assessed. | 4 |
| Outcome | [#6b](https://www.goodreports.org/reporting-checklists/tripod/info/#6b) | Report any actions to blind assessment of the outcome to be predicted. | NA |
| Predictors | [#7a](https://www.goodreports.org/reporting-checklists/tripod/info/#7a) | Clearly define all predictors used in developing or validating the multivariable prediction model, including how and when they were measured | 4-5 |
| Predictors | [#7b](https://www.goodreports.org/reporting-checklists/tripod/info/#7b) | Report any actions to blind assessment of predictors for the outcome and other predictors. | NA |
| Sample size | [#8](https://www.goodreports.org/reporting-checklists/tripod/info/#8) | Explain how the study size was arrived at. | 5 |
| Missing data | [#9](https://www.goodreports.org/reporting-checklists/tripod/info/#9) | Describe how missing data were handled (e.g., complete-case analysis, single imputation, multiple imputation) with details of any imputation method. | 5 |
| Statistical analysis methods | [#10a](https://www.goodreports.org/reporting-checklists/tripod/info/#10a) | If you are developing a prediction model describe how predictors were handled in the analyses. | Supplementary table 2 |
| Statistical analysis methods | [#10b](https://www.goodreports.org/reporting-checklists/tripod/info/#10b) | If you are developing a prediction model, specify type of model, all model-building procedures (including any predictor selection), and method for internal validation. | 5-6 |
| Statistical analysis methods | [#10c](https://www.goodreports.org/reporting-checklists/tripod/info/#10c) | If you are validating a prediction model, describe how the predictions were calculated. | 6 |
| Statistical analysis methods | [#10d](https://www.goodreports.org/reporting-checklists/tripod/info/#10d) | Specify all measures used to assess model performance and, if relevant, to compare multiple models. | 6 |
| Statistical analysis methods | [#10e](https://www.goodreports.org/reporting-checklists/tripod/info/#10e) | If you are validating a prediction model, describe any model updating (e.g., recalibration) arising from the validation, if done | 6 |
| Risk groups | [#11](https://www.goodreports.org/reporting-checklists/tripod/info/#11) | Provide details on how risk groups were created, if done. | NA |
| Development vs. validation | [#12](https://www.goodreports.org/reporting-checklists/tripod/info/#12) | For validation, identify any differences from the development data in setting, eligibility criteria, outcome, and predictors. | 6 |
| **Results** |  |  |  |
| Participants | [#13a](https://www.goodreports.org/reporting-checklists/tripod/info/#13a) | Describe the flow of participants through the study, including the number of participants with and without the outcome and, if applicable, a summary of the follow-up time. A diagram may be helpful. | 6-7 and figure 1 |
| Participants | [#13b](https://www.goodreports.org/reporting-checklists/tripod/info/#13b) | Describe the characteristics of the participants (basic demographics, clinical features, available predictors), including the number of participants with missing data for predictors and outcome. | Table 1 and Supplementary table 2 |
| Participants | [#13c](https://www.goodreports.org/reporting-checklists/tripod/info/#13c) | For validation, show a comparison with the development data of the distribution of important variables (demographics, predictors and outcome). | Table 1 |
| Model development | [#14a](https://www.goodreports.org/reporting-checklists/tripod/info/#14a) | If developing a model, specify the number of participants and outcome events in each analysis. | 6 |
| Model development | [#14b](https://www.goodreports.org/reporting-checklists/tripod/info/#14b) | If developing a model, report the unadjusted association, if calculated between each candidate predictor and outcome. | NA |
| Model specification | [#15a](https://www.goodreports.org/reporting-checklists/tripod/info/#15a) | If developing a model, present the full prediction model to allow predictions for individuals (i.e., all regression coefficients, and model intercept or baseline survival at a given time point). | Table 2 |
| Model specification | [#15b](https://www.goodreports.org/reporting-checklists/tripod/info/#15b) | If developing a prediction model, explain how to the use it. | 10 and supplementary figure 1 |
| Model performance | [#16](https://www.goodreports.org/reporting-checklists/tripod/info/#16) | Report performance measures (with CIs) for the prediction model. | 9 |
| Model-updating | [#17](https://www.goodreports.org/reporting-checklists/tripod/info/#17) | If validating a model, report the results from any model updating, if done (i.e., model specification, model performance). | NA |
| **Discussion** |  |  |  |
| Limitations | [#18](https://www.goodreports.org/reporting-checklists/tripod/info/#18) | Discuss any limitations of the study (such as nonrepresentative sample, few events per predictor, missing data). | 12-13 |
| Interpretation | [#19a](https://www.goodreports.org/reporting-checklists/tripod/info/#19a) | For validation, discuss the results with reference to performance in the development data, and any other validation data | NA |
| Interpretation | [#19b](https://www.goodreports.org/reporting-checklists/tripod/info/#19b) | Give an overall interpretation of the results, considering objectives, limitations, results from similar studies, and other relevant evidence. | 10-13 |
| Implications | [#20](https://www.goodreports.org/reporting-checklists/tripod/info/#20) | Discuss the potential clinical use of the model and implications for future research | 11 |
| **Other information** |  |  |  |
| Supplementary information | [#21](https://www.goodreports.org/reporting-checklists/tripod/info/#21) | Provide information about the availability of supplementary resources, such as study protocol, Web calculator, and data sets. | Supplementary material |
| Funding | [#22](https://www.goodreports.org/reporting-checklists/tripod/info/#22) | Give the source of funding and the role of the funders for the present study. | 13 |

None The TRIPOD checklist is distributed under the terms of the Creative Commons Attribution License CC-BY. This checklist can be completed online using <https://www.goodreports.org/>, a tool made by the [EQUATOR Network](https://www.equator-network.org) in collaboration with [Penelope.ai](https://www.penelope.ai)

NA=not applicable;

Supplementary table 2. Assignment and missing data of candidate predictors

| Candidate predictors | Assignment |
| --- | --- |
| Age at baseline | NA |
| Sex |  |
|  | 1=male |
|  | 2=female |
| Residence |  |
|  | 0=city |
|  | 1=town |
|  | 2=rural |
| Education |  |
|  | 0= illiteracy |
|  | 1= primary school |
|  | 2= secondary school and above |
| QOL |  |
|  | 0= very good |
|  | 1=good |
|  | 2=general |
|  | 3=bad |
| Exercise |  |
|  | 0=no |
|  | 1=yes |
| Physical disability |  |
|  | 0=no |
|  | 1=yes |
| Marriage |  |
|  | 0= unmarried |
|  | 1= married |
|  | 2= widowed |
| BMI |  |
|  | 0= normal |
|  | 1= underweight |
|  | 2= overweight |
|  | 3= obesity |
| Sleep |  |
|  | 0= good |
|  | 1=general |
|  | 2=poor |
| Smoke |  |
|  | 0=no |
|  | 1=yes |
| Drink |  |
|  | 0=no |
|  | 1=yes |

QOL=quality of life; BMI=body mass index

Supplementary table 3. The characteristics of the participants between those with missing data and complete data.

| Variables | **Participants with complete data** | | **Participants with missing data** | | ***P value*** |
| --- | --- | --- | --- | --- | --- |
|  | **Number of participants in each variable** | **median (IQR) / n (%)** | **Number of participants in each variable** | **median (IQR) / n (%)** |  |
| **Age (year)** | 2420 | 76.00 (71.00, 83.00) | 2420 | 76.00 (71.00, 83.00) | 1.000 |
| **Sex** | 2420 |  | 2420 |  | 1.000 |
| Male |  | 1215 (50.2) |  | 1215 (50.2) |  |
| Female |  | 1205 (49.8) |  | 1205 (49.8) |  |
| **Residence** | 2420 |  | 2420 |  | 1.000 |
| City |  | 1380 (57.0) |  | 1380 (57.0) |  |
| Town |  | 323 (13.3) |  | 323 (13.3) |  |
| Rural |  | 717 (29.6) |  | 717 (29.6) |  |
| **Education** | 2420 |  | 2420 |  | 1.000 |
| Illiteracy |  | 1129 (46.7) |  | 1129 (46.7) |  |
| Primary school |  | 933 (38.6) |  | 933 (38.6) |  |
| Secondary school or above |  | 358 (14.8) |  | 358 (14.8) |  |
| **QOL** | 2420 |  | 2406 |  | 1.000 |
| Very good |  | 433 (17.9) |  | 430 (17.9) |  |
| Good |  | 1039 (42.9) |  | 1037 (43.1) |  |
| General |  | 845 (34.9) |  | 837 (34.8) |  |
| Bad |  | 103 (4.3) |  | 102 (4.2) |  |
| **Exercise** | 2420 |  | 2348 |  | 0.894 |
| No |  | 138 (5.7) |  | 136 (5.8) |  |
| Yes |  | 2282 (94.3) |  | 2212 (94.2) |  |
| **Physical disability** | 2420 |  | 1546 |  | <0.001 |
| No |  | 1997 (82.5) |  | 1523 (98.5) |  |
| Yes |  | 423 (17.5) |  | 23 (1.5) |  |
| **Marriage** | 2420 |  | 2380 |  | 0.993 |
| Married |  | 1397 (57.7) |  | 1391 (57.8) |  |
| Widowed |  | 994 (41.1) |  | 989 (41.1) |  |
| Unmarried |  | 29 (1.2) |  | 28 (1.2) |  |
| **BMI** | 2420 |  | 2390 |  | 1.000 |
| Underweight |  | 359 (14.8) |  | 355 (14.9) |  |
| Normal |  | 1351 (55.8) |  | 1332 (55.7) |  |
| Overweight |  | 524 (21.7) |  | 518 (21.7) |  |
| Obesity |  | 186 (7.7) |  | 185 (7.7) |  |
| **Sleep** | 2420 |  | 2420 |  | 1.000 |
| Good |  | 1535 (63.4) |  | 1535 (63.4) |  |
| General |  | 599 (24.8) |  | 599 (24.8) |  |
| Poor |  | 286 (11.8) |  | 286 (11.8) |  |
| **Smoke** | 2420 |  | 2404 |  | 0.986 |
| Yes |  | 530 (21.9) |  | 526 (21.9) |  |
| No |  | 1890 (78.1) |  | 1878 (78.1) |  |
| **Drink** | 2420 |  | 2392 |  | 0.973 |
| Yes |  | 516 (21.3) |  | 511 (21.4) |  |
| No |  | 1904 (78.7) |  | 1881 (78.6) |  |

IQR=interquartile range; n=number; QOL=quality of life; BMI=body mass index

Supplementary table 4. The importance of the six predictors in cognitive frailty prediction model.

| **Remove variables** | **McFadden pseudo-R^2^** | **Difference value** |
| --- | --- | --- |
| Age | 0.15332 | 0.07882 |
| Sex | 0.22941 | 0.00273 |
| BMI | 0.21759 | 0.01455 |
| Residence | 0.22956 | 0.00258 |
| Exercise | 0.22996 | 0.00218 |
| Physical disability | 0.18383 | 0.04831 |

Full model pseudo-R^2^: 0.23214

Supplementary figure 1. The nomogram for predicting cognitive frailty
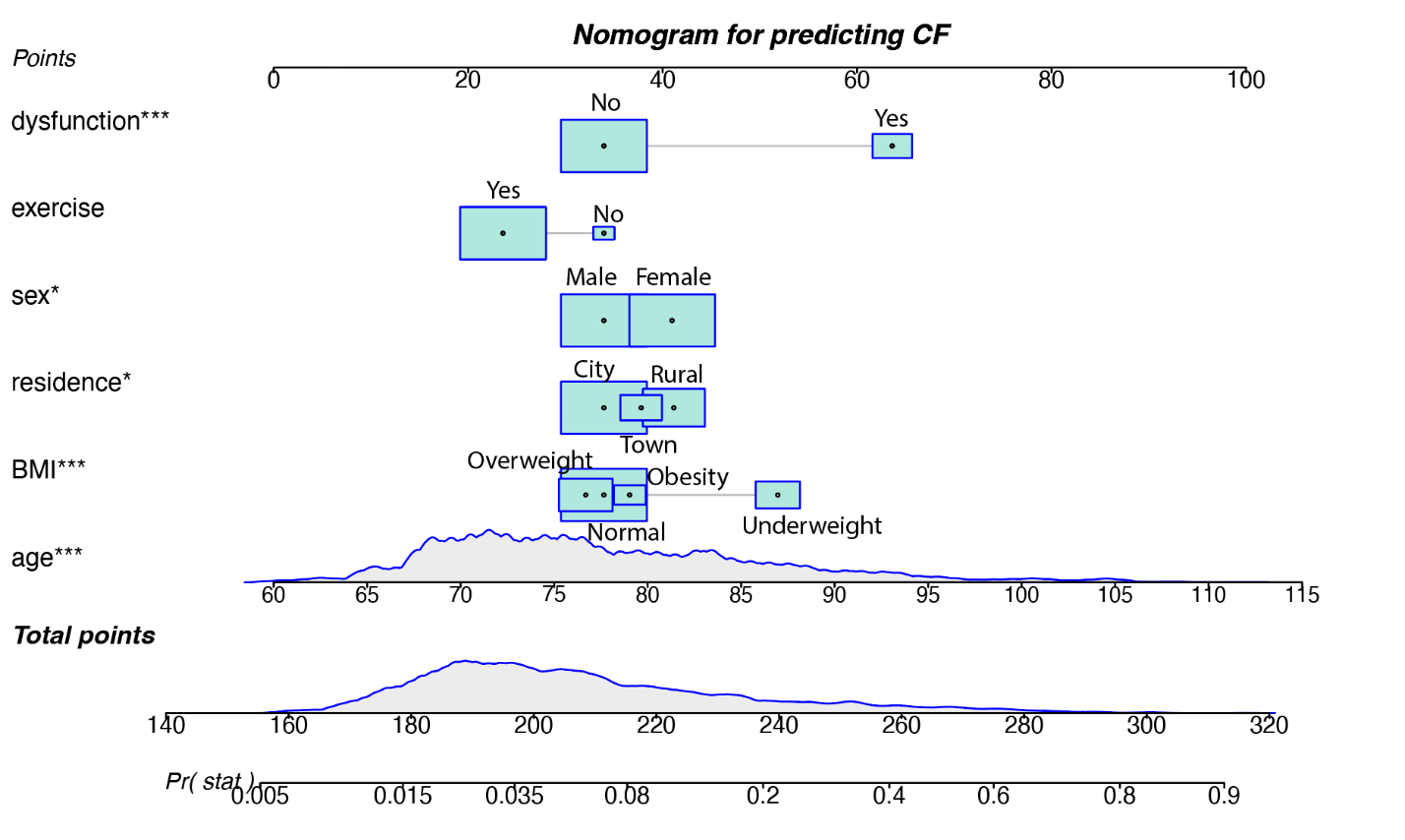


Supplementary figure 2. The ROC of prediction model excluding BMI.


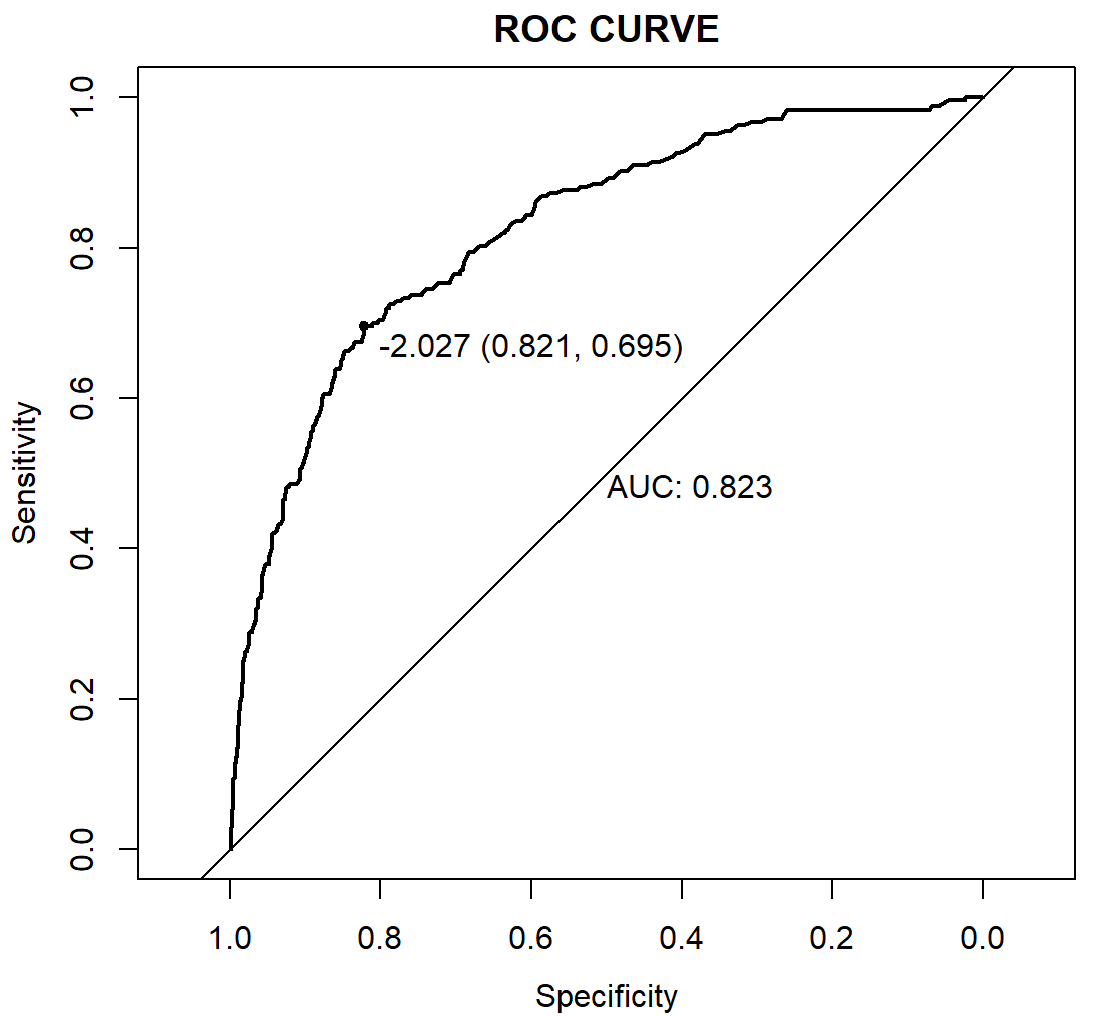


Supplementary figure 3. The ROC of prediction model for participants without missing data.


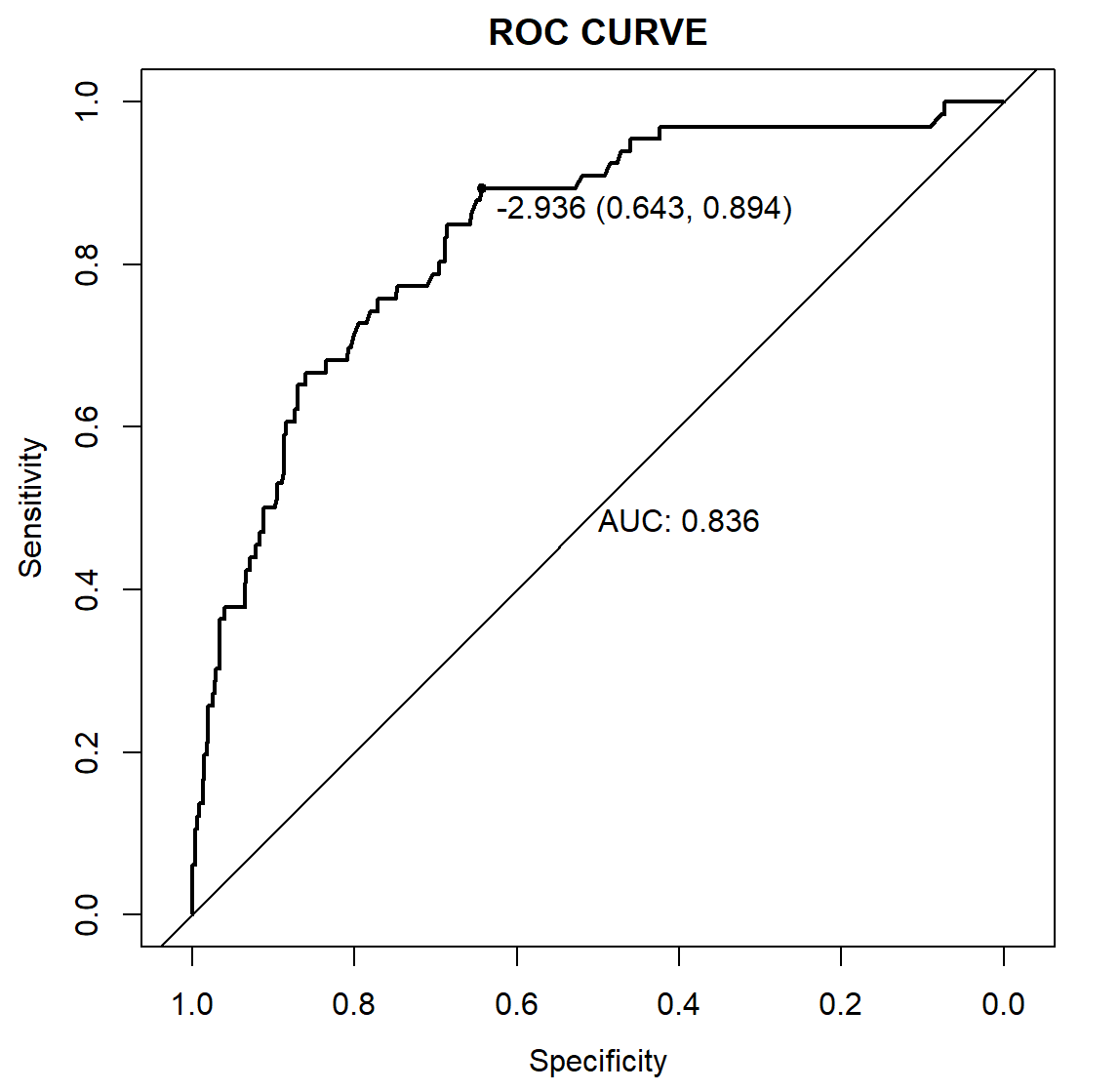

Supplement: Supplementary file 1 — Supplementary file1 (DOCX 215 KB) [file 40520_2023_2647_MOESM1_ESM.docx]
